# ctDNA to Predict Treatment Response in Head and Neck Squamous Cell Carcinoma: A Systematic Review

**DOI:** 10.1002/lary.32440

**Published:** 2025-07-17

**Authors:** Oliver Jones, Sumrit Bola, Stuart C. Winter

**Affiliations:** ^1^ Medical Student, School of Medicine and Biomedical Sciences, Medical Sciences Division University of Oxford Oxford UK; ^2^ Consultant Head and Neck Surgeon, Blenheim Head and Neck Unit Oxford University NHS Foundation Trust Oxford UK; ^3^ Consultant Head and Neck Surgeon, Nuffield Department of Surgical Sciences, Medical Sciences Division University of Oxford Oxford UK

**Keywords:** ctDNA, head and neck, HNSCC, prognostication, surveillance

## Abstract

**Objective:**

To investigate the potential role of plasma circulating tumor DNA (ctDNA) in predicting treatment response in HPV‐positive and HPV‐negative Head and Neck Squamous Cell Carcinoma (HNSCC).

**Data Sources:**

Medline, PubMed, Cochrane databases (Cochrane Library, Cochrane Central Register of Controlled Trials) and EMBASE databases were searched in accordance with the standard PICOTS model.

**Review Methods:**

Search results were independently reviewed by two primary authors. Disagreements were adjudicated by the senior author. Studies were excluded according to defined exclusion criteria. Studies were assessed for quality in accordance with the REMARK guidelines. Data were then extracted, and findings were discussed narratively.

**Results:**

From a total of 1026 articles initially retrieved, 18 papers were included in the final review. Study quality was suboptimal, with a median REMARK score of 12.65/20. Short follow‐up (median 24.5 months) and modest cohort sizes (median 59 patients) were observed. Most studies (13/18) investigated HPV ctDNA alone, and ctDNA detection methods varied considerably between studies. Broadly, studies showed that ctDNA monitoring has a high negative predictive value (91.7%) for residual and recurrent disease. Persistently positive ctDNA corresponded with poorer outcomes overall, but there was variability in the positive predictive value between studies, which was mitigated by consecutive testing.

**Conclusion:**

These results suggest there is potential for ctDNA utility in surveillance for both HPV‐positive and HPV‐negative HNSCC patients; however, better adherence to reporting guidelines will enable meta‐analysis.

## Graphical abstract

This systematic review aims to investigate the role of plasma circulating tumor DNA in head and neck cancer surveillance. We conducted a systematic review to comprehensively assess the findings and quality of existing literature on this topic. Our findings show that ctDNA monitoring post‐treatment likely has utility in HNSCC surveillance. It could be hoped that our findings will inform future studies and meta‐analysis to further answer the question as to whether ctDNA monitoring after treatment could improve outcomes in head and neck cancer.

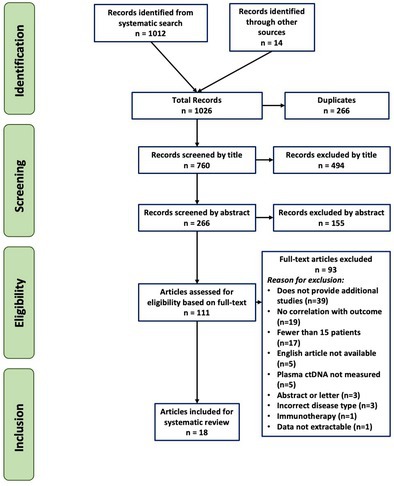

## 1 Introduction

Head and neck squamous cell carcinoma (HNSCC) represents a global health burden as the seventh most common cancer worldwide [1]. Patient outcomes vary significantly based on human papillomavirus (HPV) status, with 5‐year overall survival rates of 50% and 80% for HPV‐negative disease and HPV‐positive disease, respectively. Disease recurrence, distant metastases, and second primary tumors confer a poor prognosis [2, 3]. In locally advanced HNSCC, recurrence rates are reported to range from 10% to 20%, with median survival declining to 6–15 months [4, 5].

HNSCC surveillance protocols primarily rely on initial post‐treatment imaging followed by clinical monitoring alone, as recommended by national guidelines [6]. This approach often results in the delayed detection of disease recurrence, as clinical manifestations may lag months behind actual tumor progression. For this reason, many research groups have investigated other techniques to improve post‐treatment surveillance.

Circulating tumor DNA (ctDNA), comprising tumor‐derived fragments detectable in peripheral blood plasma, has emerged as a promising biomarker for monitoring persistent solid tumors [7, 8, 9]. Advanced molecular techniques, including polymerase chain reaction (PCR) and next‐generation sequencing (NGS), enable detection, characterization, and quantification of ctDNA, with potential for rapid clinical implementation [10, 11]. However, the utility of ctDNA in HNSCC remains unknown and recent research has rapidly expanded, with previous work summarizing its potential role in surveillance and early diagnosis of HNSCC [12, 13].

This systematic review identifies recent studies investigating the role of ctDNA in HNSCC surveillance and is the first to follow recommendations for tumor MARKer prognostic studies (REMARK) guidelines to assess study quality [14]. The analysis evaluates the capability of plasma ctDNA to detect (minimal) residual disease and recurrence in both HPV‐positive and HPV‐negative patients after treatment for laryngeal, oropharyngeal, or hypopharyngeal cancer.

## 2 Methods

This study was registered and approved by PROSPERO (ID: CRD42023442143). Since registration, minor amendments to the protocol were made to incorporate a wider breadth of available literature following a deeper understanding of the relative shortage of appropriate studies.

This review was reported in accordance with PRISMA 2020 (Data S1).

### 2.1 Search Strategy

Medline, PubMed, Cochrane databases (Cochrane Library, Cochrane Central Register of Controlled Trials) and EMBASE databases were searched in August 2023 and repeated in August 2024 using combinations of the phrases:

“head and neck cancer, head and neck carcinoma, head cancer, larynx cancer, mouth cancer, neck cancer, nose cancer, pharynx cancer, tongue cancer, tonsil cancer, palatal cancer, disease progression, recurrence, ctDNA, cfDNA” (Data S2).

### 2.2 Selection Criteria

This systematic review was designed in concordance with the PICOTS model [15]. The population of interest included patients with HNSCC, specifically oropharyngeal, laryngeal, and hypopharyngeal disease. The intervention under investigation was post‐treatment plasma ctDNA monitoring, which was compared to standard of care surveillance methods including imaging and clinical examination. The primary outcomes of interest were detection of disease recurrence and minimal residual disease. The timing encompassed studies published between 2013 and 2024, with a required median follow‐up of at least 9 months. Where follow‐up was not explicitly reported, an estimate was calculated from data presented. The setting focused on post‐treatment clinical monitoring in patients who received curative‐intent treatment. Studies were excluded if they had follow‐up data for fewer than 15 patients or lacked a verified English translation. Letters, abstracts, and case reports were also excluded. For multiple publications from the same patient cohort, the study that best met inclusion criteria was selected.

Publications were independently reviewed and collated by two primary authors, with disagreements resolved by the senior author. When published data were unclear, corresponding authors were contacted and given 6 months to provide clarification.

### 2.3 Data Extraction

Publication titles were screened for appropriateness, with inappropriate titles and duplicates excluded. Remaining papers were screened by abstract for appropriateness; the full‐text was reviewed if one author selected an abstract. Papers were obtained for full review and data extraction. Survival, cohort size, methodology, treatment modality, and outcomes were independently extracted by two authors.

### 2.4 Quality Assessment

The quality of each study was subjectively assessed against the “Reporting recommendations for tumor MARKer prognostic studies” (REMARK) guidelines. Papers were subjectively red, amber, green (RAG)‐rated according to 20 criteria, and a numerical score was given to each paper. Scoring methodology was performed using a framework from Iafolla et al. [16]. Scoring details from Iafolla et al. can be found in Data S3.

In addition to REMARK scoring, risk of bias was assessed using the QUADAS‐2 tool.

### 2.5 Statistical Analysis

Where appropriate, data provided within the main article or Supporting Information was used to calculate sensitivity, specificity, positive predictive value (PPV) and negative predictive value (NPV) and was compared with the published values if available. If there were discrepancies between the reported and calculated data, authors were contacted for clarification.

Analysis was performed using Microsoft Excel and statistical software: GraphPad Prism v10.4.1 and R v4.3.1.

## 3 Results

### 3.1 Search Results

**FIGURE 1:**
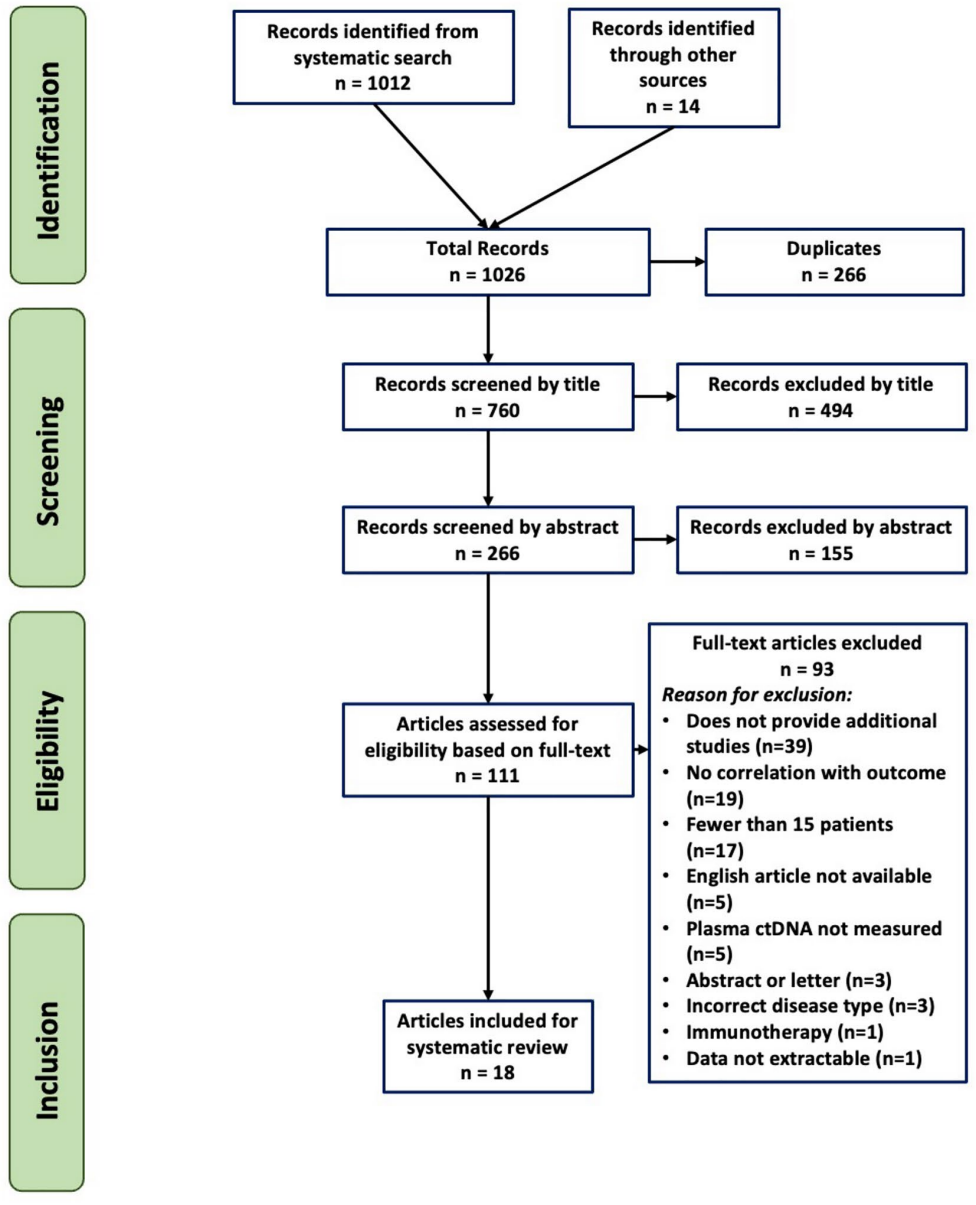
PRISMA flow diagram. Reasons for full‐text exclusions are depicted above. [Color figure can be viewed in the online issue, which is available at www.laryngoscope.com]

A total of 1026 articles were initially identified. 760 articles remained after de‐duplication. After title and abstract screening, 111 articles remained for full‐text review. 18 articles ultimately fulfilled the inclusion criteria (Figure 1).

#### 3.1.1 Study Methodology and Patient Characteristics

Included papers were published between 2013 and 2024. The median sample size was 59 patients (Range = 18–1076; total = 2447). Of the 11 studies that reported median age, the overall median age was 63 (Range = 26–92). Of the 12 papers that reported a median follow‐up time for the cohort, the overall median follow‐up time was 24.5 months (0.9–106 months). Where clinical follow‐up was not stated, data were used to estimate a median time from treatment to ensure inclusion criteria was met. The median REMARK score was 12.65 (Range = 8.91–15.48), out of a theoretical maximum of 20 (Data S4).

An assessment of the risk of bias using the QUADAS‐2 assessment found that blinding information was rarely given, and patient flow was often difficult to manage across most studies. To gain large numbers, multiple sites had to be involved, which introduced potential bias and different methods assessing the reference standard. Regardless, the applicability of results across the domains was consistently good. The findings of this assessment are shown in Table 1.

**TABLE 1:** QUADAS‐2 assessment.

Study	Patient selection		Index test		Reference standard		Flow and timing
	R	A	R	A	R	A	R
Ahn (2014)	High	Low	Low	Low	Low	Low	High
Berger (2022)	Low	Low	Low	Low	High	Low	Unclear
Bergener (2021)	High	Low	High	Low	Low	Low	Low
Campo (2024)	Low	Low	Low	Low	Low	Low	High
Cao (2022)	Low	Low	Low	Low	Low	Low	Unclear
Chera (2020)	Low	Low	Low	Low	Low	Low	Low
Dietrich (2023)	Low	Low	Low	Low	Low	Unclear	Low
Ferrandino (2023)	High	Low	Unclear	Low	Low	Low	Low
Honoré (2023)	Low	Low	Low	Low	Low	Low	Unclear
Kogo (2022)	Low	Low	Low	Low	Low	Low	High
Lee (2017)	Low	Low	High	Low	Low	Low	Low
O'Boyle (2022)	Low	Low	High	Low	Low	Unclear	Unclear
Roof (2024)	Unclear	Low	Unclear	Low	Low	Low	Low
Routman (2022)	High	Low	Low	Low	Low	Low	High
Rutkowski (2020)	Low	Low	Unclear	Low	Low	Low	High
Tanaka (2021)	Low	Low	Unclear	Low	Low	Low	Low
Warlow (2022)	Low	Low	Unclear	Low	Low	Low	High
Wu (2021)	High	Low	Low	Low	Low	Low	Low

Abbreviations: A, applicability concerns; R, risk.

Eight studies were conducted in the United States, two in Japan, two in the United Kingdom, and one in Canada, Italy, Germany, Belgium, Poland, and China. Most studies (13/18; 72%) focused on HPV ctDNA alone. Three (17%) investigated the detection of somatic ctDNA only, and two (11%) investigated both somatic and HPV ctDNA. All papers investigating HPV ctDNA (*n* = 15) targeted HPV‐16, eight also targeted HPV‐18, seven targeted HPV‐31, and eight targeted HPV‐33 and 35 (Table 2). In a more recent review of the literature (February 2025), a correction was noted for Chera et al. 2020. This correction was due to patient consent, rather than data accrual, and did not change the conclusions of that study or this review [35].

**TABLE 2:** A summary of included studies.

Study	Disease and treatment	Patients and follow‐up	Assay	Key results	Limitations
Ahn (2014) [17] (Retrospective) United States	OPSCC Unknown primary HPV+ for analysis (Surgery or RT/CRT)	52 had HPV+ tumors Follow‐up 1–181 months (median 49)	qPCR for HPV16 E6, E7, B‐Actin	35 ctDNA+ pre‐treatment 8/52 had recurrence, of which 3 tested negative HPV‐patient was ctDNA+ post‐treatment with no recurrence High Specificity Median lead time 4.4 months	No temporal relationship between ctDNA positivity and recurrence Low sensitivity
Berger (2022) [18] (Retrospective) United States	OPSCC HPV+ (Curative intent treatment)	1076 Follow‐up 6–22 months (median 9)	TTMV‐HPVDNA for HPV‐16, 18, 31, 33, 35	TTMV‐HPVDNA positivity preceded clinical confirmation of recurrence in 59/80 PPV and NPV were 95%	Short follow‐up Threshold for positivity arbitrarily set at > 7 for HPV‐16 and > 12 for HPV‐18, 31, 33, 35
Burgener (2021) [19] (Prospective) Canada	Stage I–IVA HPV‐ (Surgery ± RT/CRT)	18 Follow‐up 7–57 months (median 41.5)	CAPP‐Seq and cfMeDIP‐seq (NGS) for somatic mutations	13/18 ctDNA− post‐treatment. 9/13 remained disease‐free Pre‐treatment ctDNA was associated with worse overall survival High specificity	Small sample Unknown outcome for 1 patient Low sensitivity
Campo (2024) [20] (Prospective) Italy	OPSCC incl. palate and unknown HPV+ for analysis (RT/CRT, surgery)	74 Follow‐up not stated	ddPCR for HPV‐16, 33, 35	49/60 HPV+ had undetectable post‐treatment ctDNA by week 10. None recurred 11/60 had ctDNA persistence or increase. Recurrence detected in 10/11	HPV‐ outcomes unreported Assumes p16 status as the standard for HPV+ diagnosis
Cao (2022) [21] (Prospective) United States	Stage III p16+ OPSCC HPV+ (CRT)	28 Follow‐up 12–49 (median 28)	ddPCR for HPV‐16, 18	Week 2 ctDNA kinetics may predict tumor recurrence Week 4 and 7 ctDNA kinetics, did not predict disease progression	Specific cohort of HNSCC Marked advertisement (18 USC Section 1734)
Chera (2020) [22] (Prospective) United States	Stage I–III p16+ HPV+ OPSCC (RT/CRT)	115 Follow‐up 6.1–54.7 months (median 23)	dPCR for HPV‐16, 18, 31, 33, 35	87 were ctDNA− post‐treatment and remained disease free (NPV = 100%) 15/28 patients ctDNA+ post CRT had recurrence Two consecutive ctDNA+ tests gave a PPV to 94%. One test PPV 54%	Missing bloods prior to recurrence in 4/15 patients Non‐uniform treatment with 97 patients having de‐escalation of IMRT (60Gy)
Dietrich (2023) [23] (Prospective) Germany	OPSCC, oral cavity, hypopharynx, larynx, nasal cavity, unknown primary HPV+ and HPV‐ (Surgery ± RT/CRT)	219 Follow‐up 1–106 months (median 27)	Methylation‐specific qRT‐PCR	Post‐treatment SEPT9 methylation was predictive of positive surgical margin and poorer DFS Specificity modest (79%)	Limited application in surveillance Sensitivity low (46%)
Ferrandino (2023) [24] (Retrospective) United States	OPSCC HPV+ (Surgery or RT/CRT)	290 Follow‐up 17–67.5 months (median 40.5)	TTMV‐HPVDNA for HPV‐16, 18, 31, 33, 35	Test specificity 100%, sensitivity 88% Median lead time 47 days (0–507) in 13 patients	Only summary outcomes reported
Honoré (2023) [25] (Prospective) Belgium	OPSCC, larynx, oral cavity, hypopharynx, unknown primary HPV + and HPV‐ (Surgery or RT/CRT)	41 Follow‐up 2.9–46.5 months (median 31)	NGS, Kraken2 for HPV 16, ddPCR for validation	14/17 ctDNA+ patients had disease progression or a non‐head and neck cancer 4/24 ctDNA− patients had recurrence	Lack of serial sampling, partly due to patient refusal Only 10 HPV‐16+ patients Low sensitivity of Kraken2 compared to ddPCR
Kogo (2022) [26] (Prospective) Japan	OPSCC, hypopharynx, larynx, external auditory canal HPV+ and HPV‐ (Surgery or CRT/RT)	18 Follow‐up 3.2–33.9 months (median18.5)	dPCR for HPV DNA and somatic mutations NGS to profile	Bespoke ctDNA panels 11/18 ctDNA− post‐treatment, no recurrence 7/18, remained positive or became positive. All relapsed. 5/7 were positive before or at the time of relapse	In 6 patients, bespoke ctDNA panels could not be designed Small sample size
Lee (2017) [27] (Prospective) United Kingdom	Stage III/IV Oropharynx, larynx, hypopharynx HPV+ for surveillance (CRT)	59 Follow‐up not stated	ddPCR and HPV detect for HPV‐16	27/27 patients with CRR were ctHPVDNA− 3/4 patients with PET‐CT findings had negative biopsy and were ctHPVDNA− 1/4 ctHPVDNA+ and recurred	Assumption that PET‐CT result is always correct Clinical follow‐up not clear, however not focus of paper
O'Boyle (2022) [28] (Retrospective) United States	OPSCC HPV+ (Surgery or CRT)	33 Follow‐up 6–20 months (median 12)	ddPCR for HPV‐16, 18, 31, 33, 35	Residual disease risk factors associated with higher POD1 ctHPVDNA One patient recurred Time from positive test to recurrence was 56 days	Short follow‐up Infrequent post‐treatment blood tests. (POD1, 7, 30, 3 months and 1 year)
Roof (2024) [29] (Retrospective) United States	OPSCC HPV+ (Surgery, CRT/RT, Immunotherapy)	210 Follow‐up not stated Estimated median 11.5 months	TTMV‐HPVDNA for HPV‐16, 18, 31, 33, 35	TTMV‐HPV DNA testing had a 97.5% accuracy in determining recurrence TTMV‐HPV DNA testing can resolve clinically indeterminate findings during surveillance	Statistics calculated from number of tests rather than patient numbers Sampling times at clinician's discretion Immunotherapy patients included in analysis No detailed follow‐up
Routman (2022) [30] (Prospective) United States	OPSCC HPV+ (Surgery)	32 Follow‐up mean 14.4 months	ddPCR for HPV‐16, 18, 31, 33, 35	2/32 recurred, both ctHPVDNA+ ctHPVDNA+ patients had 18 month RFS of 83% compared to 100% for ctHPVDNA− patients Post‐operative ctHPVDNA was associated with RFS	Inconsistent time‐points for blood sampling
Rutkowski (2020) [31] (Prospective) Poland	OPSCC HPV+ (CRT/RT)	66 Follow‐up not stated. At least 12 months	qPCR for HPV‐16	1/43 was ctDNA+ initially. Later became ctHPVDNA− with no recurrence 5/23 with IRR were ctHPVDNA+. 18/23 were ctHPVDNA negative and did not recur	Timings of serial blood sampling after 12 weeks not clear
Tanaka (2021) [32] (Prospective) Japan	Oropharynx, larynx, hypopharynx, unknown primary HPV+ (CRT/RT)	35 Follow‐up 7–36 months (median 21)	ddPCR for HPV‐16	ctHPVDNA has a similar NPV, but a greater PPV to post‐treatment PET‐CT ctHPVDNA− patients has 12 month DFS of 33% compared to 93% for ctHPVDNA− patients	Comparisons to PET‐CT made at the end of study, rather than at 3‐months
Warlow (2022) [33] (Prospective) United Kingdom	OPSCC HPV+ for analysis (Surgery, CRT/RT, Immunotherapy)	48 Median follow‐up 20 months	ddPCR for HPV‐16, 18, 31, 33, 35	40/48 were ctDNA− post treatment, all remained disease‐free 8/48 were ctDNA+ and 7/8 recurred or had disease progression Variable clearance rates	Short follow up 1 palliative patient not included in longitudinal analysis
Wu (2021) [34] (Prospective) China	Oral cavity, Hypopharynx, Larynx HPV‐ (Surgery ± RT/CRT)	27 Follow‐up 1.3–27.2 months (median 20.7 months)	NGS for somatic mutations	6/8 who recurred were ctDNA+ post‐treatment Post‐operative ctDNA+ patients had shorter DFS than ctDNA− patients Large gene panel	Surveillance not the focus of this paper Small sample size

Abbreviations: CRR, Complete radiological response; CRT, Chemoradiotherapy; ctHPVDNA−, ctHPVDNA negative; ctHPVDNA+, ctHPVDNA positive; DFS, disease‐free survival; IRR, Incomplete radiological response; OPSCC, Oropharyngeal squamous cell carcinoma; POD1, post‐operative day 1; RFS, recurrence‐free survival; RT, radiotherapy.

Of the HPV‐negative studies, Burgener et al. [19] and Dietrich et al. [23] assessed methylation patterns, and Kogo et al. [26] assessed somatic variants in the primary tumor and designed personalized NGS panels for each patient. Wu et al. [34] primarily conducted a mutational profiling study of tumor tissue, blood, and saliva but used a non‐personalized panel to detect somatic variants post‐treatment. Honoré et al. also used an NGS sequencing panel of the most frequently mutated HNSCC genes but had a smaller panel than Wu et al. which included 2 HPV‐16 genes.

A summary of detection methods and study quality is described in Data S5.

#### 3.1.2 Post‐Treatment ctDNA as a Marker of Disease

The association between post‐treatment ctDNA detection and a defined clinical endpoint was assessed to a varying extent in all 18 studies.

##### 3.1.2.1 HPV Positive Disease

Thirteen studies evaluated ctDNA in HPV‐positive disease alone, with follow‐up periods ranging from 0.9 to 181 months and cohort sizes varying from 18 to 1076 patients. Studies reporting diagnostic metrics for ctHPVDNA detection demonstrated robust performance characteristics, with mean sensitivity and specificity of 80.2% and 97%, respectively (PPV = 86.7%, NPV = 95.8%) (Table 3). Test‐specific performance metrics can be found in Data S6.

**TABLE 3:** Basic statistics of the effectiveness of post‐treatment ctDNA measuring after treatment to predict clinical outcome in studies assessing patients with HPV positive disease. NB: Values stated in Chera et al. are those following two consecutive tests.

Study	Test	Sensitivity	Specificity	PPV	NPV
Tanaka	ddPCR	66.7%*	100%*	100%	89.7%
Campo	ddPCR	100%	98.4%	90.9%	100%
Routman	ddPCR	69.2%*	78.1%*	22%*	96.7%*
Warlow	ddPCR	70%*	97.4%*	92.5%*	87.5%*
Berger	TTMV‐HPV DNA	55%*	100%*	95%	95%
Ferrandino	TTMV‐HPV DNA	88.4%	100%	100%	99.1%
Roof	TTMV‐HPV DNA	90.5%*	100%*	100%*	96.8%*
Ahn	qPCR	62.5%*	97.7%*	83.3%*	93.5%*
Rutkowski	qPCR	100%	98%	83%	100%
Chera	dPCR	100%	100%	100%	100%
O'Boyle	ddPCR	Not reported	Not reported	Not reported	Not reported
Cao	ddPCR	Not reported	Not reported	Not reported	Not reported
Lee	HPV detect	Not reported	Not reported	Not reported	Not reported

* Value calculated by authors based on in‐text data.

O'Boyle and colleagues conducted a prospective investigation of ctHPVDNA clearance kinetics in the immediate post‐operative period following transoral robotic surgery. Their analysis of 12 surgical patients revealed that persistent detection at post‐operative day one correlated with residual disease burden (macroscopic or microscopic); therefore, it informed early adjuvant treatment decision‐making. Patients with macroscopic residual disease had significantly higher ctHPVDNA compared to those patients without pathological risk factors (*p* = 0.0037). Notably, four clinically low‐risk patients who achieved ctHPVDNA clearance within 6 h remained disease‐free, suggesting that rapid clearance may indicate complete tumor resection.

In a subgroup analysis by Lee et al. a notable case demonstrated the potential superiority of ctHPVDNA over conventional imaging. Despite negative clinical examination and PET‐CT findings at 12 weeks, persistently elevated ctHPVDNA levels preceded radiological detection of recurrence by 18 months. Conversely, three patients with PET‐positive findings but negative biopsies maintained undetectable ctHPVDNA levels, with subsequent imaging confirming the absence of disease.

Warlow and colleagues, utilizing ddPCR, highlighted that the emergence of detectable ctHPVDNA following initial clearance strongly predicted disease recurrence. All 40 patients maintaining undetectable ctHPVDNA remained disease‐free, and 7/8 patients with persistent or increasing ctDNA levels developed recurrence. Clearance was achieved in 39/40 patients by the first blood sample (mean = 10 weeks, range = 2–22 weeks).

Campo et al. documented five cases where ctHPVDNA levels, initially undetectable post‐treatment, showed subsequent elevation either preceding or coinciding with confirmed disease progression. Similarly, Rutkowski et al. reported that among 23 patients with radiological evidence of residual disease at 12‐week PET‐CT, only 5 demonstrated positive ctHPVDNA by qPCR, all of whom had confirmed disease on subsequent investigation. All 18 ctHPVDNA‐negative patients showed no evidence of disease on biopsy, yielding a negative predictive value of 100%. One case demonstrated delayed ctHPVDNA clearance without subsequent recurrence, suggesting potential variability in molecular remission kinetics as seen in Warlow et al.

In a subset of 7 patients under intensive ctDNA monitoring, Chera and colleagues found that 8% had a transient rise in ctHPVDNA that resolved spontaneously without disease recurrence. This led to the proposed dual‐positive criterion for recurrence prediction, where two consecutive tests improved the positive predictive value from 50% to 100%, although these parameters were retrospectively derived. Their longitudinal monitoring observed a median lead time of 6.6 months between a positive ctHPVDNA result and clinical diagnosis for recurrence.

Interestingly, Cao et al. observed that early ctDNA elevation at week 2 post‐treatment correlated with improved progression‐free survival in p16+ OPSCC patients undergoing CRT, potentially reflecting treatment‐induced DNA shedding. In contrast to other studies, they found no prognostic value in ctDNA levels at weeks 4–7 post‐treatment.

##### 3.1.2.2 HPV‐Negative Disease

Studies reporting diagnostic metrics for ctDNA detection in HPV‐negative HNSCC (Table 4) demonstrated a mean sensitivity and specificity of 62.5% and 88.5%, respectively (PPV = 77.1%, NPV = 80.3%). Two papers investigated the role for ctDNA testing in combination with HPV‐positive disease [25, 26], and three in HPV‐negative patients alone [19, 23, 34]. Sample size varied from 18 to 219, and follow‐up ranged from 1 to 106 months.

**TABLE 4:** Basic statistics of the effectiveness of post‐treatment ctDNA measuring after treatment to predict clinical outcome in studies assessing somatic mutations or methylation for HPV‐negative disease.

Study	Test	Sensitivity	Specificity	PPV	NPV
Kogo	dPCR	71.4%*	100%*	84.6%*	100%*
Dietrich	Methylation‐specific qRT‐PCR	46%*	79.3%*	39.7%	83.2%
Honoré	NGS	82.4%	78.6%	82.3%	78.6%
Burgener	CAPP‐seq and cfMeDIP‐seq	50%*	90%*	80%*	69.2%*
Wu	NGS	62.5%	94.7%	83.3%*	85.7%*

* Value calculated by authors based on in‐text data.

Kogo and colleagues employed tumor‐informed sequencing to develop personalized ctDNA monitoring panels. In their cohort, 7 of 18 cases demonstrated either post‐treatment ctDNA reemergence following initial clearance or persistent detection. All seven cases corresponded with either disease recurrence or residual tumor burden. Notably, no recurrences were observed among the 11 patients maintaining undetectable ctDNA levels throughout the follow‐up period (median = 18.5 months).

Wu et al. also utilized NGS for post‐treatment ctDNA detection, identifying eight relapses, six of whom were ctDNA‐positive. Interestingly, they detected a *CDKN2A* mutation in ctDNA that was absent in primary tumor tissue, highlighting potential limitations of tumor‐informed ctDNA panel approaches, as proposed by Kogo et al., despite their favorable performance metrics.

Similarly, Honoré et al. developed a ctDNA panel targeting 26 frequently mutated genes identified through literature review. While their methodology assumed post‐treatment ctDNA detection indicated residual disease, and some variants were classified as unknown significance or benign, the prognostic value was evident. Patients with detectable post‐treatment ctDNA had significantly reduced 2‐year progression‐free survival (23.53%) compared to ctDNA‐negative cases (86.6%).

Burgener and colleagues implemented dual molecular approaches (CAPP‐seq and cfMeDIP‐seq) for mutation and aberrant methylation detection in 18 patients. Their analysis showed that failure to achieve ctDNA clearance correlated with increased recurrence risk. Among 13 patients achieving undetectable post‐treatment ctDNA levels, nine remained disease‐free, while four recurred. Five patients (28%) demonstrated no clearance of ctDNA after treatment, which was associated with an increased risk of recurrence.

Dietrich and colleagues used an entirely different approach and investigated *SEPT9* methylation as a prognostic biomarker using methylation‐specific quantitative RT‐PCR. Their findings established post‐surgical *SEPT9* ctDNA positivity as an independent prognostic factor for HNSCC (HR: 2.43, *p* = 0.008), albeit with comparatively modest sensitivity, specificity, positive predictive value, and negative predictive value metrics (Table 4).

#### 3.1.3 Surgery Versus CRT

The performance of ctDNA in detecting disease was compared based on primary treatment modality. Five studies analysed patients who underwent curative‐intent surgery (± RT/CRT) [19, 23, 28, 30, 34]. The mean sensitivity and specificity of this group were 57% and 85.5%, respectively (PPV = 56.3%, NPV = 83.7%).

In the non‐surgical cohort, six studies evaluated patients receiving primary CRT or RT alone [21, 22, 27, 28, 31, 32]. These investigations exclusively focused on detecting ctHPVDNA in HPV‐positive patients. This cohort demonstrated superior diagnostic performance, with mean sensitivity and specificity of 88.9% and 99.3% respectively (PPV = 94.3%, NPV = 96.6%).

Eight additional studies incorporated mixed treatment modalities in their analysis [17, 18, 20, 24, 25, 26, 29, 33], limiting discrete evaluation of the predictive capability of ctDNA between the two modalities.

#### 3.1.4 Comparison to Current Post‐Treatment Surveillance

Multiple papers reported ctDNA positivity as the earliest indicator of disease recurrence, preceding both radiological and clinical diagnosis [18, 24, 25, 29]. However, the time interval between molecular and conventional detection varied greatly (lead time range 0–507days [24]), and some sample sizes were small, with two studies only reporting 1 recurrence [27, 28]. In Tanaka et al., the NPV and PPV for the ctDNA HPV test were 89.7% and 100%, respectively, compared to the corresponding metric of PET‐CT in this cohort (NPV 84%, PPV 50%). While ctDNA detection post‐treatment does not universally predict recurrence, the cumulative evidence suggests that it represents a promising early molecular indicator of residual or recurrent disease.

## 4 Discussion

Recent therapeutic advances in HNSCC treatment have underscored the need for improved post‐treatment surveillance and the early identification of disease. This review suggests that plasma ctDNA represents a promising surveillance modality. While previous systematic reviews have highlighted the potential of ctHPVDNA as an ideal biomarker, this comprehensive critical appraisal of existing literature provides additional insights into the role of ctDNA in HNSCC monitoring in HPV‐positive and HPV‐negative patients.

Methodological assessment revealed that most studies had modest cohort sizes and sub‐optimal follow‐up duration (median = 24.5 months) representing less than half of the standard 5‐year clinical surveillance window. Poor adherence to REMARK guidelines (Data S4) complicated analysis, with variation in sampling protocols and clinical endpoints. Only nine of the 18 studies reported performance statistics, limiting the potential for meaningful meta‐analyses [36]. Regardless, the QUADAS‐2 tool demonstrated that applicability concerns were generally low, and so poor scoring does not necessarily reflect the relevance of the studies. Consistent with previous reviews, investigations predominantly focused on HPV‐positive disease [12, 37], reflecting the technical feasibility of detecting circulating viral HPV DNA compared to somatic mutations or methylation analysis. In particular, the TTMV‐HPV DNA assay demonstrated a high positive predictive value in HPV‐positive patients.

Post‐treatment ctDNA monitoring demonstrated consistently high NPV in HPV positive patients, supporting its utility in excluding residual or recurrent disease [36]. Three patients in the Lee et al. cohort with concerning radiological imaging but negative ctDNA had negative biopsies, suggesting that ctDNA outperformed routine radiological imaging. Further investigations may be warranted to determine whether negative ctDNA results could inform personalized adjuvant treatment strategies. One potential application is the modification of surveillance intensity for patients who remain ctDNA negative. However, the occurrence of false‐negative results across several cohorts necessitates careful consideration, and de‐intensification protocols would need to be paired with patient education and open communication, like the PETNECK2 trial protocol [38]. While negative results generally correlate with improved outcomes, the transition from undetectable to detectable ctDNA strongly correlated with disease recurrence, emphasizing the importance of regular molecular monitoring during follow‐up.

The utility of ctHPVDNA monitoring in post‐treatment surveillance relied on two fundamental assumptions: that HPV‐positive tumors consistently release HPV DNA, and that HPV infection in the absence of malignancy does not generate detectable circulating viral DNA. These assumptions remain incompletely validated, and although the false‐positive rates were low, a separate study identified three female volunteers in the control arm (non‐HNSCC group) had circulating HPV‐16 [39], raising questions against this hypothesis. Overall, the positive predictive value of ctHPVDNA monitoring was variable, likely reflecting multiple factors, including the potential of concurrent HPV infection or cervical HPV disease. However, without longer follow‐up, it is difficult to ascertain if the positive ctHPVDNA test represented molecular disease.

In HPV‐negative patients, emerging evidence showed promise. Kogo et al. demonstrated the feasibility of tumor‐informed mutation panels with reported sensitivity and specificity of 71.4% and 100%, respectively [26]. However, emerging somatic mutations [34], cost, tissue availability, and clonal evolution may compromise personalized ctDNA panel utility. Dietrich et al. investigated *SEPT9* methylation as a biomarker for disease. A comparatively low NPV and PPV suggested that alone, it did not have potential to detect recurrence, but a bivariate analysis showed that when combined with AJCC/UICC tumor stage, *SEPT9* methylation was an independent prognostic factor for HNSCC. Collectively, the studies exploring molecular surveillance in HPV‐negative disease established promising foundational evidence for further investigation.

Treatment modality influenced ctDNA dynamics. O'Boyle et al. found that persistent ctDNA correlated with residual disease in surgical cohorts, while Cao and colleagues observed that elevated early ctDNA levels in chemoradiotherapy patients are associated with improved outcomes. This paradoxical finding in the chemoradiotherapy cohort may reflect treatment‐induced tumor cell death and subsequent DNA release. These contrasting observations suggest that treatment affects molecular kinetics, which potentially impacts personalized decision‐making for adjuvant therapy.

Among studies that employed frequent post‐treatment ctDNA measurements after initial treatment, select patients had transient rises in ctDNA with no observed recurrence. This was an unexplained dynamic contributing to false‐positive results but mitigated by a repeat test, as demonstrated by Chera et al., where two consecutive ctDNA tests significantly enhanced the PPV.

Comparative analysis showed superior PPV for ctDNA monitoring in CRT/RT versus surgical cohorts, though study heterogeneity limits definitive conclusions.

## 5 Limitations

The findings of this review are subject to publication bias, and the exclusive focus on plasma ctDNA precludes evaluation of salivary ctDNA, potentially valuable in oral cavity malignancies [40]. Additionally, variations in reporting, follow‐up, and detection methodologies limited direct comparison between many studies.

## 6 Conclusion

Current literature provides compelling evidence for ctDNA utility in head and neck squamous cell carcinoma management, both as a prognostic tool and for early detection of recurrence or minimal residual disease. However, this review identifies a notable bias toward HPV‐positive disease investigation. Given the substantially worse prognosis associated with recurrent HPV‐negative disease, there exists a critical need for large‐scale studies evaluating the role of ctDNA in this population. Present evidence remains insufficient for clinical implementation, attributable to the relative novelty and paucity of high‐quality published data. Future investigations should adhere to REMARK guidelines where applicable, facilitating standardization of results and enabling comprehensive evaluation of post‐treatment ctDNA monitoring. Large‐scale prospective studies are essential to definitively establish optimal ctDNA‐guided surveillance protocols, including evaluation of sequential testing strategies. Regardless, the potential for ctDNA utility in surveillance was clear across all studies for both surgically and non‐surgically treated patients.

## Conflicts of Interest

The authors declare no conflicts of interest.

## Supporting information


**Data S1.** PRISMA Checklist.

**Data S2.** Detailed search strategy.

**Data S3.** REMARK Scoring framework replicated from Iafolla et al.

**Data S4.** Study quality assessed using the “Reporting recommendations for tumor MARKer prognostic studies” (REMARK) guidelines. Each study was independently assessed using the 20 criteria outlined in the REMARK guidelines. Green circles indicate the criterion was fully satisfied, orange circles indicate partially satisfied, and red circles indicate the criterion was not satisfied at all. REMARK scores out of a possible 20 are also represented. Higher REMARK scores represent studies of greater quality.

**Data S5.** Schematic showing the method of ctDNA detection for each study grouped based on whether they detected HPV ctDNA, somatic ctDNA, or both.

**Data S6.** Summary statistics for studies investigating HPV‐positive disease.
