# Supplementary material for: ctDNA to Predict Treatment Response in Head and Neck Squamous Cell Carcinoma: A Systematic Review
Source: Laryngoscope. 2025 Jul 17;136(1):50–62. doi: 10.1002/lary.32440 (PMC12770799; doi:10.1002/lary.32440)
Supplement: Supplementary file 2 — Data S2. Detailed search strategy. [file LARY-136-50-s004.docx]

Supplementary Data 2. Detailed search strategy.

**1**

"head and neck cancer"/ or "head and neck carcinoma"/ or head cancer/ or larynx cancer/ or lip cancer/ or mouth cancer/ or neck cancer/ or nose cancer/ or pharynx cancer/ or salivary gland cancer/ or tongue cancer/ or tonsil cancer/ or palatal cancer/

**2**

((Head or Neck* or Oral or Pharyn* or Oropharyn* or Hypopharyn* or Laryn* or tongue or Nasal* or Cheek* or lip* or tonsil* or sino-nasal or palat*) and (carcinoma* or neoplasm* or cancer* or metastas* or tumo?r* or Squamous Cell Carcinoma*)).mp. [mp=title, abstract, heading word, drug trade name, original title, device manufacturer, drug manufacturer, device trade name, keyword heading word, floating subheading word, candidate term word]

**3**

HNSCC.mp.

**4**

1 or 2 or 3

**5**

(Predict* or Detect*).mp. [mp=title, abstract, heading word, drug trade name, original title, device manufacturer, drug manufacturer, device trade name, keyword heading word, floating subheading word, candidate term word]

**6**

recurrent disease/

**7**

(Relaps* or Recur*).mp. [mp=title, abstract, heading word, drug trade name, original title, device manufacturer, drug manufacturer, device trade name, keyword heading word, floating subheading word, candidate term word]

**8**

6 or 7

**9**

4 and 5 and 8

**10**

(ctDNA or (circulating adj4 DNA) or cell-free or cell free DNA or cfDNA).mp. [mp=title, abstract, heading word, drug trade name, original title, device manufacturer, drug manufacturer, device trade name, keyword heading word, floating subheading word, candidate term word]

**11**

9 and 10
