# Supplementary material for: ctDNA to Predict Treatment Response in Head and Neck Squamous Cell Carcinoma: A Systematic Review
Source: Laryngoscope. 2025 Jul 17;136(1):50–62. doi: 10.1002/lary.32440 (PMC12770799; doi:10.1002/lary.32440)
Supplement: Supplementary file 3 — Data S3. REMARK Scoring framework replicated from Iafolla et al. [file LARY-136-50-s001.docx]

**Supplementary Data 3.** REMARK Scoring framework replicated from Iafolla *et al.*

| **REMARK criteria number** | **Criteria number used in score** | **Criteria description** | **Potential points awarded** |
| --- | --- | --- | --- |
| 1 | 1 | Marker stated. | 0.33 |
|  | 2 | Objective stated. | 0.33 |
|  | 3 | Pre-specified hypothesis stated. | 0.33 |
| 2 |  |  |  |
|  | 4 | Source of patients. | 0.33 |
|  | 5 | Inclusion criteria (i.e. stage of cancer). | 0.33 |
|  | 6 | Exclusion criteria. | 0.33 |
|  | 7 | If applicable: how specific cases were included if drawn from a parent study. | 0.25 |
| 3 | 8 | Details of treatment. | 0.5 |
|  | 9 | Timing of therapy relative to specimen collection. | 0.5 |
| 4 |  |  |  |
|  | 10 | Methods of preservation. | 0.33 |
|  | 11 | Storage. | 0.33 |
|  | 12 | Time between time of storage and time of marker assay. | 0.33 |
|  | 13 | If applicable: if controls are used, then details on the control’s morbidities, medications, sex, age, etc. | 0.25 |
| 5 |  |  |  |
|  | 14 | Amount of specimen required to perform the assay. | 0.33 |
|  | 15 | Strategies employed to reduce the measurement error. | 0.33 |
|  | 16 | Blinding of the person making the marker assessment to clinical outcomes. | 0.33 |
|  | 17 | If applicable: multicentre studies must state if single reviewers or reference laboratories are used to reduce variability in marker measurements. | 0.25 |
| 6 |  |  |  |
|  | 18 | Time period cases were taken. | 0.25 |
|  | 19 | The end of follow-up period. | 0.25 |
|  | 20 | Median follow-up time. | 0.25 |
|  | 21 | Marker measurements were extracted retrospectively from existing records, assays were newly performed using stored specimens, or assays were performed in real time using prospectively collected specimens. | 0.25 |
|  | 22 | If applicable: patients were stratified by clinicopathologic factors. | 0.20 |
| 7 | 23 | The endpoint should be defined precisely. | 1 |
| 8 | 24 | Fully define all variables. | 1 |
| 9 | 25 | Either sample size calculation, or effect size calculation given the pre-determined sample size. | 1 |
| 10 |  |  |  |
|  | 26 | Describe statistical methods with sufficient detail for verification. | 0.5 |
|  | 27 | Must state that “all data was accounted for” or “no missing data occurred”. | 0.5 |
| 11 | 28 | For continuous variables: clarify whether the data were kept on the original scale or log transformed, and indicate whether the relationship was modelled as linear or non-linear. For categorized variables: specify the cutpoints and how they were chosen. | 1 |
| 12 |  |  |  |
|  | 29 | The study must show either a flow diagram (e.g. CONSORT), or a study profile diagram. | 0.5 |
|  | 30 | Report the number of patients and the number of events. | 0.5 |
| 13 | 31 | Distributions of basic demographic variables and standard prognostic variables. | 0.5 |
|  | 32 | Description of the distribution of the marker of interest. | 0.5 |
| 14 |  |  |  |
|  | 33 | The association of the tumour marker with standard prognostic variables. | 1 |
| 15 | 34 | Univariable relation between a categorical marker and outcome. | 0.33 |
|  | 35 | Univariable confidence intervals. | 0.33 |
|  | 36 | Univariable P-value. | 0.33 |
| 16 | 37 | Multivariable relation between a categorical marker and outcome. | 0.33 |
|  | 38 | Multivariable confidence intervals. | 0.33 |
|  | 39 | Multivariable P-value. | 0.33 |
| 17 | 40 | The study must evaluate whether the new marker maintains some association with clinical outcome after accounting for these standard prognostic variables. | 0.25 |
|  | 41 | Confidence intervals. | 0.25 |
|  | 42 | P-value. | 0.25 |
|  | 43 | Discussion and explanation of how these standard variables have been selected. | 0.25 |
| 18 | 44 | Must test their model with one of the following: test their assumption, sensitivity analysis, or internal validation analyses or external validation studies. | 1 |
| 19 | 45 | One of the following: acknowledgment of any biases or inconsistencies in the data, limitations of the assay methods, or limitations of the design or data analysis methods. | 0.5 |
|  | 46 | Comment on whether the results are consistent with, or differ from, the general tendency in previous studies and offer potential explanations for differences. | 0.5 |
| 20 | 47 | A discussion if the biomarker is clinically useful. | 0.5 |
|  | 48 | Future research plans. | 0.5 |
