# Supplementary material for: ctDNA to Predict Treatment Response in Head and Neck Squamous Cell Carcinoma: A Systematic Review
Source: Laryngoscope. 2025 Jul 17;136(1):50–62. doi: 10.1002/lary.32440 (PMC12770799; doi:10.1002/lary.32440)
Supplement: Supplementary file 4 — Data S4. Study quality assessed using the “Reporting recommendations for tumor MARKer prognostic studies” (REMARK) guidelines. Each study was independently assessed using the 20 criteria outlined in the REMARK guidelines. Green circles indicate the criterion was fully satisfied, orange circles indicate partially satisfied, and red circles indicate the criterion was not satisfied at all. REMARK scores out of a possible 20 are also represented. Higher REMARK scores represent studies of greater quality. [file LARY-136-50-s003.docx]

**Supplementary Data 4.** Study quality assessed using the `Reporting recommendations for tumour MARKer prognostic studies` (REMARK) guidelines. Each study was independently assessed using the 20 criteria outlined in the REMARK guidelines. Green circles indicate the criterion was fully satisfied, orange circles indicate partially satisfied, and red circles indicate the criterion was not satisfied at all. REMARK scores out of a possible 20 are also represented. Higher REMARK scores represent studies of greater quality.


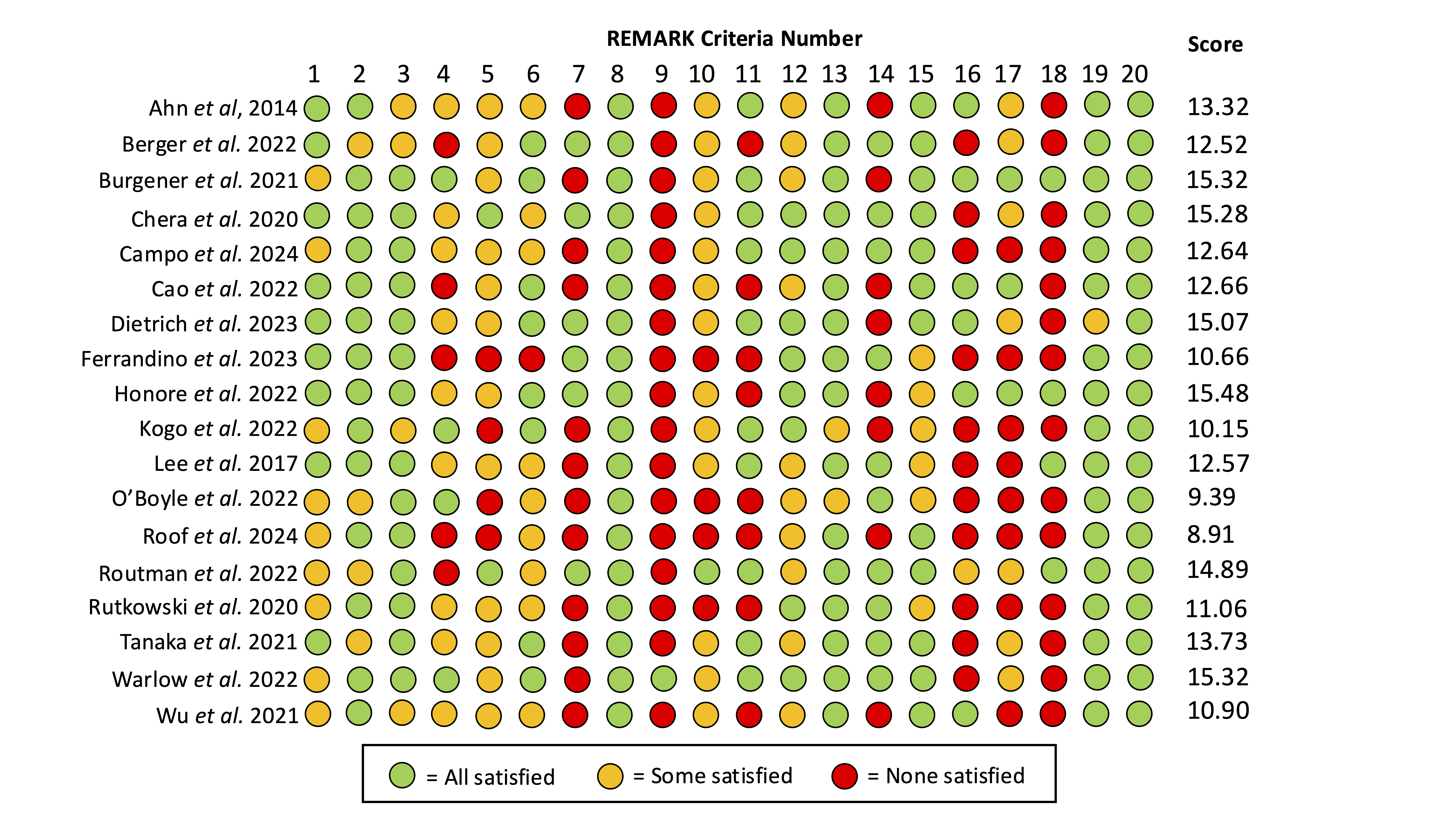


The median REMARK score was 12.77 (Range=8.91-15.48), out of a theoretical maximum of 20. The highest scoring was Honoré *et al*. 2022, and the lowest scoring was Roof *et al.* 2024. Patient cohort sizes were generally small (median = 59), and one study performed a sample/effect size calculation to determine the minimum sample size required (REMARK criteria 9)^34^. Additionally, only six studies precisely defined an endpoint and only four only performed internal or external validation (REMARK criteria 18). Sensitivity, specificity, positive predictive value, and negative predictive value of longitudinal ctDNA monitoring were reported in 9 of the 18 included studies (Table 3A, B).

REMARK scores were normally distributed (Shapiro Wilk, 0.915, p = 0.11) and there was no correlation between year of publication and overall REMARK score (Pearson correlation, r = -0.65, p = 0.53).
