# Supplementary material for: ctDNA to Predict Treatment Response in Head and Neck Squamous Cell Carcinoma: A Systematic Review
Source: Laryngoscope. 2025 Jul 17;136(1):50–62. doi: 10.1002/lary.32440 (PMC12770799; doi:10.1002/lary.32440)
Supplement: Supplementary file 5 — Data S5. Schematic showing the method of ctDNA detection for each study grouped based on whether they detected HPV ctDNA, somatic ctDNA, or both. [file LARY-136-50-s005.docx]

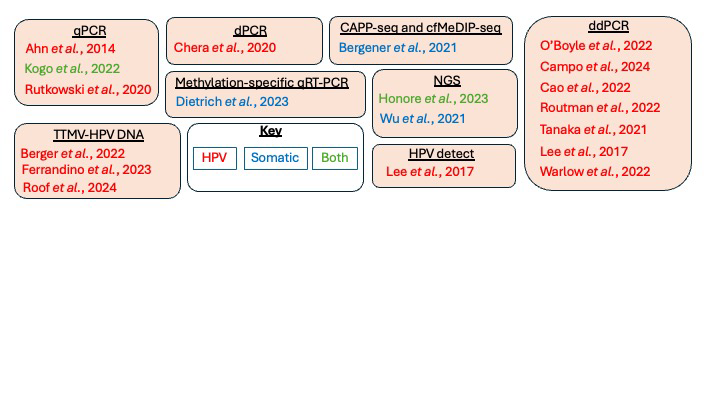


**Supplementary Data 5**. Schematic showing the method of ctDNA detection for each study grouped based on whether they detected HPV ctDNA, somatic ctDNA, or both.
