# Supplementary material for: ctDNA to Predict Treatment Response in Head and Neck Squamous Cell Carcinoma: A Systematic Review
Source: Laryngoscope. 2025 Jul 17;136(1):50–62. doi: 10.1002/lary.32440 (PMC12770799; doi:10.1002/lary.32440)
Supplement: Supplementary file 6 — Data S6. Summary statistics for studies investigating HPV‐positive disease. [file LARY-136-50-s006.docx]

***Supplementary Data 6: Summary statistics for studies investigating HPV-positive disease***

Six of these papers detected HPV ctDNA using ddPCR, of which four reported test performance metrics (Table 3). The mean sensitivity, specificity was 76.5% and 93.5% respectively (PPV=76.4%, NPV=93.5%). Two papers used qPCR to detect ctDNA, with a mean sensitivity and specificity of 81.3% and 97.9%, respectively (PPV= 83.2%, NPV=96.75%). Three papers used a TTMV-HPV DNA assay, with a mean sensitivity and specificity of 78.0% and 100%, respectively (PPV=98.3%, NPV=97%). Chera *et al.* used dPCR where the calculated sensitivity, specificity for two consecutive tests 100% and 99%, respectively (PPV=94%, NPV=100%).
